# Clonal hematopoiesis and its impact on the aging osteo-hematopoietic niche

**DOI:** 10.1038/s41375-024-02226-6

**Published:** 2024-03-21

**Authors:** Susann Winter, Katharina S. Götze, Judith S. Hecker, Klaus H. Metzeler, Borhane Guezguez, Kevin Woods, Hind Medyouf, Alexander Schäffer, Marc Schmitz, Rebekka Wehner, Ingmar Glauche, Ingo Roeder, Martina Rauner, Lorenz C. Hofbauer, Uwe Platzbecker

**Affiliations:** 1 Department of Internal Medicine I, University Hospital Carl Gustav Carus, Faculty of Medicine Carl Gustav Carus, TU Dresden, Dresden, Germany; 2 German Cancer Consortium (DKTK), CHOICE Consortium, Partner Sites Dresden/Munich/Frankfurt/Mainz, and German Cancer Research Center (DKFZ), Heidelberg, Germany; 3 Department of Medicine III, Technical University of Munich (TUM), School of Medicine and Health, Munich, Germany; 4 German MDS Study Group (D-MDS), Leipzig, Germany; 5 TranslaTUM, Center for Translational Cancer Research, Technical University of Munich (TUM), Munich, Germany; 6 Department of Hematology, Cellular Therapy, Hemostaseology and Infectious Disease, University of Leipzig Medical Center, Leipzig, Germany; 7 Department of Hematology and Oncology, University Medical Center Mainz, Mainz, Germany; 8 Institute for Tumor Biology and Experimental Therapy, Georg-Speyer-Haus, Frankfurt am Main, Germany; 9 Frankfurt Cancer Institute, Frankfurt am Main, Germany; 10 Institute of Immunology, Faculty of Medicine Carl Gustav Carus, TU Dresden, Dresden, Germany; 11 National Center for Tumor Diseases (NCT), Dresden, Germany: German Cancer Research Center (DKFZ), Heidelberg, Germany; Faculty of Medicine and University Hospital Carl Gustav Carus, TU Dresden, Dresden, Germany; Helmholtz-Zentrum Dresden–Rossendorf (HZDR), Dresden, Germany; 12 Institute for Medical Informatics and Biometry, Faculty of Medicine Carl Gustav Carus, TU Dresden, Dresden, Germany; 13 Division of Endocrinology, Diabetes and Bone Diseases, Department of Medicine III, and Center for Healthy Aging, University Medical Center, TU Dresden, Dresden, Germany

**Keywords:** Haematopoietic stem cells, Cancer microenvironment, Translational research, Haematological cancer

## Abstract

Clonal hematopoiesis (CH) defines a premalignant state predominantly found in older persons that increases the risk of developing hematologic malignancies and age-related inflammatory diseases. However, the risk for malignant transformation or non-malignant disorders is variable and difficult to predict, and defining the clinical relevance of specific candidate driver mutations in individual carriers has proved to be challenging. In addition to the cell-intrinsic mechanisms, mutant cells rely on and alter cell-extrinsic factors from the bone marrow (BM) niche, which complicates the prediction of a mutant cell’s fate in a shifting pre-malignant microenvironment. Therefore, identifying the insidious and potentially broad impact of driver mutations on supportive niches and immune function in CH aims to understand the subtle differences that enable driver mutations to yield different clinical outcomes. Here, we review the changes in the aging BM niche and the emerging evidence supporting the concept that CH can progressively alter components of the local BM microenvironment. These alterations may have profound implications for the functionality of the osteo-hematopoietic niche and overall bone health, consequently fostering a conducive environment for the continued development and progression of CH. We also provide an overview of the latest technology developments to study the spatiotemporal dependencies in the CH BM niche, ideally in the context of longitudinal studies following CH over time. Finally, we discuss aspects of CH carrier management in clinical practice, based on work from our group and others.

## Introduction

During aging, dividing cells and tissues, including hematopoietic stem cells (HSCs) that give rise to mature blood and immune cells, can acquire somatic mutations in cancer-associated genes. If such a mutation confers a selective growth advantage, this particular mutant HSC clone can progressively expand over time within specific BM niches and lead to CH. Recent studies uncovered cigarette smoking, male sex, and longer leukocyte telomere length as risk factors to acquire mutations in HSCs [1–3]. Genetic variants in several germline loci have also been identified as inheritable determinants predisposing to CH [3, 4]. In 2015, the term “clonal hematopoiesis of indeterminate potential” (CHIP) was introduced to distinguish nonmalignant CH that is linked to the presence of candidate driver mutations associated with hematologic malignancies from other forms of CH [5]. Such clonal expansion becomes increasingly detectable with age with divergent health implications [6, 7]. CHIP is associated with an increased risk of developing hematologic malignancies (estimated hazard ratio (HR) > 10), most commonly myelodysplastic neoplasms (MDS) or acute myeloid leukemia (AML), although the absolute risk for progression to hematologic malignancies is low (annual incidence: approximately 0.5–1% of CHIP carriers) and shows large inter-individual variability [6, 8–10]. Recent studies have shown that individuals with CH exposed to chemo- or radiotherapy for a primary cancer are at an increased risk of developing secondary therapy-related myeloid malignancies [11, 12]. In addition, CHIP is associated with adverse outcomes and age-related diseases beyond hematologic malignancies, including cardiovascular disease (CVD) [6, 13], type 2 diabetes mellitus [6], osteoporosis [14], gout [15], chronic liver disease [16], and autoimmune disease [17]. Conceptually, it is conceivable that increased inflammation is both a cause and a consequence of premalignant CH [18]. In this regard, CH may pose as a common denominator behind several inflammatory diseases of the elderly and may be seen as a biomarker of unhealthy human aging. Certain age-related diseases seem to be exempt from this, as recent data unexpectedly demonstrated that CHIP carriers had a reduced risk of Alzheimer’s disease [19].

While the advent of more sensitive next-generation sequencing (NGS) assays combined with large-scale mutational profiling of population-based cohorts has helped to define the mutational landscape of CH, the spatiotemporal dependencies in the aging BM tissue itself are only beginning to emerge. BM cells function as a unit to regulate and sustain hematopoiesis and bone remodeling throughout life. Mutant hematopoietic stem and progenitor cells (HSPCs) and their progeny bear the potential to alter BM cell function via both cell-autonomous and non-autonomous mechanisms. Vice versa, changes in the aging BM microenvironment might instigate CH. This review aims to increase the consideration of the osteo-hematopoietic niche as an intricate and affected partner involved in the progression to hematologic malignancy and development of CH-associated non-malignant disease.

## Defining and detecting the spectrum of CH

In a broad sense, the term “clonal hematopoiesis” describes a clonal alteration of the hematopoietic system, and therefore would encompass all types of hematologic neoplasms. More specifically, the term CH is now used to describe the detectable outgrowth of a clonal population of circulating blood cells in individuals without known hematopoietic neoplasm or unexplained cytopenia. Initial studies in large cohorts identified CH in approximately 10–15% of elderly individuals (≥70 years), with a strong age dependency [6, 7]. These studies also showed that the known driver variants used to identify CH clones mostly affected genes also mutated in myeloid neoplasms, such as MDS and AML. In particular, gene mutations in *DNMT3A*, *TET2*, and *ASXL1* (the “DTA” genes) were identified as the most common known driver variants in CH. Based on these and other early studies, a working definition of CHIP was formulated [5], which forms the basis of current diagnostic criteria for CHIP now defined in the 5^th^ edition of the WHO classification of hematopoietic neoplasms [20]. Accordingly, a diagnosis of CHIP requires detection of ≥1 somatic mutation with a VAF ≥ 2% in blood or BM cells (≥4% for X-linked gene mutations in men) based on a specific list of mostly myeloid neoplasm-associated driver genes, as well as the absence of unexplained cytopenias or any defined myeloid neoplasm. Patients who have a clonal alteration and unexplained cytopenia, but otherwise do not meet the criteria for another hematologic cancer, would be classified as having clonal cytopenia of uncertain significance (CCUS).

While having clear diagnostic criteria delineating CHIP as an entity will be helpful in future clinical and correlative studies, it became apparent that detection of CH depends on both the scope and sensitivity of the assay used to detect clonal alterations. Therefore, it is important to realize that our understanding of CH has broadened beyond these initial narrow definitions. First, by using sensitive methods to detect variants in myeloid driver genes at VAFs below the 2% threshold, CH can be detected in over half of persons ≥70 years [17, 21]. Second, whole-exome sequencing approaches in large cohorts revealed that CH can also affect driver genes implicated in lymphoid neoplasia, thereby identifying lymphoid CH (L-CH) as a more rare cousin of myeloid (M)-CH [22]. These analyses also showed that mosaic chromosomal alterations (mCAs), and not only small-scale gene mutations, can define M-/L-CH clones. Finally, even more comprehensive genome-wide analyses of single HSCs have now shown that CH indeed becomes near-universal in humans beyond the age of approximately 70 years [23]. While hematopoiesis in young adults is highly polyclonal, these studies have revealed that in older persons, 10–20 co-existing clones that originated/expanded early in life typically account for 30–60% of total hematopoiesis. Notably, while these clones can be identified through shared somatic gene mutations, most of them do not carry any of the known myeloid or lymphoid driver variants that would be detected by targeted sequencing assays currently used in the clinic [24]. This can be seen as a further indication that even neutral clonal dynamics have an intrinsic tendency for oligo- and monoclonal conversion [25]. Overall, when discussing clinical implications of CH, it is imperative to keep in mind that hematopoiesis becomes clonal in most, or maybe all, elderly persons. In this setting, our ability to detect CH in a certain population is determined by the characteristics of the assay and the diagnostic criteria used to define clonality as much as by the underlying incidence, mutation spectrum and size of hematopoietic clones in that population. Along these lines, cross-study comparisons of CH prevalence and its clinical relevance must be viewed with great caution as differences in laboratory methods and definitions may lead to a widely different recognition of the true underlying clonal structure of hematopoiesis in study participants.

Moreover, testing for CH is typically done in bulk populations containing a mixture of different cell types with mutated and wild-type cells, limiting our ability to link genotype to phenotype. Therefore, an important goal in the field is to delineate repartition patterns and clonal dynamics of different driver variants within the hematopoietic differentiation tree. Ultimately, this will help to resolve the phenotypic and clinical consequences of CH. CH may directly or indirectly influence the differentiation capacity and/or function of various hematopoietic cell types, including neutrophils, monocytes, monocyte-derived macrophages, NK cells, B and T cells, as well as other immune-modulatory cells contributing to the osteo-hematopoietic niche, such as megakaryocytes and osteoclasts [14, 26–28]. Recent studies have shed light on the lineage involvement of the most frequently mutated “DTA” genes [21, 29, 30]. Across these studies, sorted T cells showed a significantly lower allelic burden of mutated “DTA” genes (i.e. *DNMT3A, TET2*, *ASXL1*) compared to other cell types. NK cells, considering the overall allelic burden, reached VAFs comparable to myeloid cell fractions and significantly higher than T and B cells, despite their common lymphoid progenitors [29]. Within the T cell compartment, VAFs for *DNMT3A* mutations were significantly higher compared to other mutations, indicating a multipotent stem cell origin for mutated *DNMT3A* [29, 30]. In this context, a recent study provided novel insights into the influence of M-CH, specifically *DNMT3A*-mutant CH, on the biology of lymphoid cells [31]. Using an improved single-cell sequencing pipeline, the authors showed that monocytes, CD4^+^ T cells, and NK cells carrying *DNMT3A* driver mutations present activated gene signatures, which in conjunction with indirect activation of wild-type cells by *DNMT3A*-mutant macrophages may promote progression of inflammatory reactions in patients [31]. With regard to *ASXL1*-mutant CH, our recent investigation suggests that specific *ASXL1* variants may show higher lymphoid compartment (T and B cell) involvement compared to other *ASXL1* variants [21]. These data, while based on a limited number of patients, also demonstrate that different variants for the same gene may show different lineage distribution patterns, adding to the complexity of CH.

## Temporal clonal trajectories

While CH is a condition defined at a single time point, the pathological potential emerges as a result of a dynamic process in which specific premalignant clones progressively marginalize unaffected hematopoiesis, giving rise to recirculating CH progeny. Retrospective analyses of large-scale longitudinal data of CH that allow for the direct assessment of clonal dynamics and its correlation with specific gene mutations suggest that clonal dynamics may emerge on much longer time scales in the order of decades rather than years [23, 24]. Although there is a great level of inter-individual variability, there are also clear and reproducible gene-specific differences [24, 32, 33]. Combining sequential clonal tracking with a phylogenetic reconstruction approach based on the accumulation of somatic mutations further allows to extrapolate clonal growth dynamics backward in time [24]. It is possible that some CH clones detectable in aged individuals were initiated and expanded early in life. For example, some *DNMT3A-*mutant clones are estimated to emerge during childhood or even *in utero* but display slower growth in old age [24, 34]. In contrast, other more proliferative clones (such as those driven by *SRSF2*^*P95H*^ or mutant *U2AF1*) emerge only later in life, are detected less often during mid-life, but become increasingly apparent at very old age. Interestingly, such clones do not seem to decelerate and are associated with a higher risk for AML [24]. In this context, our recent work provides an alternative in vitro culturing approach combined with computational modeling to study differentiation and proliferation kinetics of HSCs in CH [35], supplementing previous snapshot [29] and longitudinal analyses [33]. Our results suggest that hematopoietic differentiation hierarchy alterations are already detectable in the premalignant CHIP state and may contribute to emerging impaired blood production. In accordance, both *DNMT3A*- and *TET2*-mutant clones were shown to expand within the HSC compartment, while *TET2*-mutant clones had a more pronounced expansion in progenitors, particularly in the myeloid lineage [30, 36]. Unexpectedly, both mutant and non-mutant HSCs from CH samples were enriched for inflammatory and aging transcriptomic signatures, implying non-cell-autonomous changes stemming from an inflammatory microenvironment [36, 37].

In addition, very little is currently known about the dynamics and mechanisms of clonal migration and distribution. These processes may also depend on specific BM niches fostering clonal expansion. Using bilateral BM samples obtained from patients undergoing simultaneous double hip replacement surgery, we have recently demonstrated that in some patients with CH, the size of individual clones varies >10-fold between different anatomical locations, with CH variants detectable in BM from one side but not the other as well as discordance between BM and peripheral blood [21]. Thus, CH clones show spatially heterogeneous involvement in BM from different anatomical locations, suggesting that initiation and expansion of CH clones or subclones may initially be a localized process, and raising the possibility of specific BM niches conducive to clonal expansion. Work from another group has recently corroborated our findings [38].

## Clinical relevance

### CH as risk factor for cancer and inflammation

The association of CH with CVD, hematologic malignancies, therapy-related myeloid neoplasms, and death are well-known clinical implications and findings across different studies have been discussed in detail elsewhere [39, 40]. Nonetheless, we would like to point out a few important aspects. It is interesting that “DTA” genes appear to confer a similar risk of coronary heart disease despite their different biology [13]. This, according to a new study, may be explained by reverse causality, i.e. atherosclerosis driving increased HSC proliferation that leads to CH expansion in these patients [41]. While these findings require further investigation, it has become clear that variants with a higher VAF or a VAF above a certain threshold are likely to be more clinically relevant and may help guide screening and prevention strategies in CH carriers [39, 42]. In the largest study to date using exome sequence data on >40,000 CHIP carriers, high-VAF carriers were found to have an elevated risk of developing blood cancer and solid tumors, including lung, prostate, and non-melanoma skin cancer [42]. Recent data also suggest that the parallel characterization of M-/L-mCAs in conjunction with somatic mutations helps in the surveillance of cancer patients at risk of developing hematologic neoplasms [43].

CH is considered to induce dysregulated or excessive immune reactions in myeloid cells, which could contribute to chronic inflammatory diseases and lead to ineffective host immune responses to infection [44]. This spurred recent investigations to address the association of CH with severe COVID-19 outcomes and other diverse types of infection. For COVID-19, contradictory results have been published, perhaps in part due to different study design, size of the patient cohort, and variant calling and definition [42, 45, 46]. The largest of these datasets analyzed >5000 health traits from the UK Biobank and found relationships between high-VAF CHIP (VAF > 10%) and severe COVID-19 outcomes [42]. Regarding other infections, solid cancer patients with CH might be at higher risk of certain infections, such as *Clostridium difficile* and *Streptococcus/Enterococcus* infections [45]. Moreover, expanded mCAs appear to confer an increased risk of diverse incident infections, including sepsis, pneumonia, digestive tract infections, and genitourinary infections [47].

### CH as risk factor for bone and joint disorders

It is surprising that relatively little is known about the relationship between CH and bone health, given that bone and marrow are two facets of the same organ and are intertwined via several endocrine, inflammatory, and ultrastructural circuits. A recent study analyzing whole-exome sequencing data of 113,641 unrelated individuals from the UK Biobank now shows that CHIP is linked to an increased risk of incident osteoporosis in humans (HR = 1.44, 95% CI: 1.22–1.72) [14]. Larger CHIP clones, in particular in *DNMT3A*, with VAFs ≥10% correlated significantly with reduced bone mineral density. Moreover, the intimate relationship between blood and bone cells seems progressively disturbed upon malignant transformation of the osteo-hematopoietic niche. This is evident in patients with MDS who have an increased risk of incident osteoporosis (HR = 1.87, 95% CI: 1.51–2.23) [48]. In line with this, structural deterioration of the bone architecture is also seen in a murine model of MDS [49]. Conversely, prevalent osteoporosis increased the risk of incident MDS in humans (HR = 1.42, 95% CI: 1.19–1.65) [48]. Our recent finding of a high prevalence of CH (50%) among patients with osteoarthritis undergoing total hip replacement further strengthens the link of CH to inflammatory processes and altered bone remodeling [17], both of which contribute to the pathogenesis of osteoarthritis. CH may worsen joint destruction by amplifying the production of IL-1β [50], a proinflammatory cytokine that has been implicated in the pathogenesis of osteoarthritis based on indirect evidence from the CANTOS trial [51, 52].

## The aging osteo-hematopoietic niche

**Fig. 1:**
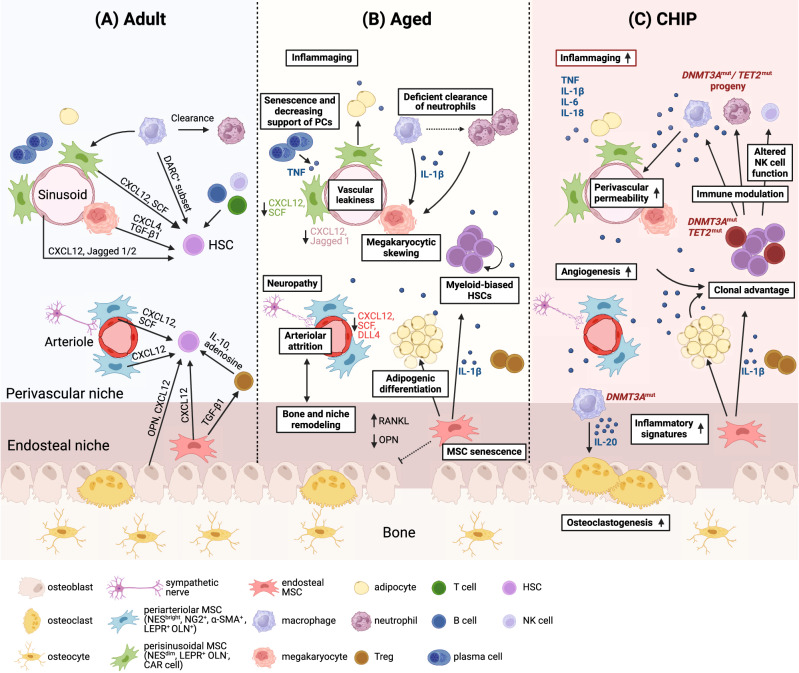
The changes in the osteo-hematopoietic niche with aging and in CHIP. **A** Various cell types and secreted niche factors directly or indirectly regulate HSC activity in the adult BM microenvironment. Periarteriolar niches localized near endosteal spaces constitute a more “dormant or quiescent” niche, specialized in promoting HSC quiescence and self-renewal. Quiescent HSCs found in these spaces associate with periarteriolar nestin^bright^ MSCs, which express the pericyte marker neural–glial antigen 2 (NG2) or the smooth muscle marker α-smooth muscle actin (α-SMA), and Schwann cells, connected to the sympathetic nervous system. Osteolineage cells, including periarteriolar LEPR^+^ osteolectin^+^ osteogenic progenitors, support the maintenance of more committed hematopoietic progenitors, in particular the lymphoid lineage. Perisinusoidal niches, comprising nestin^dim^ cells that overlap with LEPR^+^ MSCs, CAR cells, and megakaryocytes, constitute a more “proliferative” niche where HSCs proliferate and migrate. CXCL12 and SCF, two key factors involved in HSC maintenance, are widely expressed in the periarteriolar/endosteal and perisinusoidal niches. HSC-derived progeny, such as macrophages, neutrophils, Tregs, and megakaryocytes can provide feedback and contribute to HSC maintenance and mobilization. **B** Age-related alterations of the BM niche that affect HSCs include changes in the vasculature, MSCs, and osteolineage cells, with concomitant altered secretion of niche factors (e.g. reduced CXCL12, SCF, Jagged 1, and OPN), increased adipogenic and decreased osteogenic differentiation of MSCs, and increased proinflammatory cytokine expression (e.g. IL-1β, IL-6), promoting myeloid and megakaryocytic differentiation skewing. Arteries, arterioles and type H vessels, which support osteogenesis, decline with age. Concomitantly, the endosteal niche is compromised with a reduction in the number of osteoblasts and OPN. Sympathetic neuropathy (through disrupted β-adrenergic signaling) has been identified as an important determinant of niche remodeling in the aging BM. **C** In individuals with CHIP, the presence of mutant myeloid progeny (i.e. monocytes, macrophages, neutrophils) in the BM microenvironment contributes to proinflammatory cytokine expression, further increasing inflammaging in the BM and activating inflammatory transcriptional programs in aged endothelial and stromal niche cells. *DNMT3A*^*mut*^ macrophages may promote osteoclastogenesis by secreting proinflammatory cytokines, including IL-20, leading to accelerated bone loss and frailty. The resulting bone resorption bias sustains the inflammatory milieu and releases growth/niche factors that may support clonal growth and aggravate CH over time. The well-described upregulation of inflammatory mediators in *TET2*^*mut*^ monocytes/macrophages may contribute to further remodeling of vascular niches. *TET2* mutations have also been shown to contribute to repressing NK cell function. Overall, CHIP-driven remodeling of supportive BM niches can facilitate immune evasion and activate survival pathways favoring malignant clonal expansion. DARC duffy antigen receptor for chemokines (also known as ACKR1), NES nestin, OLN osteolectin, OPN osteopontin, PCs plasma cells. This image was created with BioRender.com.

The BM microenvironment provides specialized niches, which regulate the balance between HSC quiescence, activation, and subsequent cell fates through soluble factors and cell contact-dependent signals [53]. At least two anatomically different HSC niches exist in the BM: the central/perivascular niche located in the inner BM and the endosteal niche located in close proximity to the bone surface. Moreover, immune cells found in perivascular regions provide an important regulatory niche [54]. These BM niches progressively change with age, which may contribute to set the stage for CH and influence different outcomes (Fig. 1).

### Inflammaging

A central hallmark of aging is inflammaging, which is defined by elevated levels of proinflammatory markers in blood and tissues in the absence of overt infection (“sterile inflammation”). Extensive or unbalanced inflammaging increases the risk for both morbidity and mortality in elderly people and may contribute to cytopenias, inadequate immune responses, and myeloid malignancy. In fact, various proinflammatory cytokines and chemokines including TNF, IL-1, IL-6, and CCL5/RANTES show increased expression in the BM of aged individuals, many of them known to promote myeloid and megakaryocytic skewing [44]. In addition, cytokines can affect HSC function indirectly via modulating the BM microenvironment, leading to secondary inflammatory signal production by niche cells [55, 56]. Thus, inflammaging may constitute an important target for overarching interventions to “rejuvenate” HSCs.

### The aging endosteal niche

The endosteal niche accounts for up to 10% of total BM, has low oxygen tension, and mainly consists of osteogenic lineage cells of various stages intermingled with adipocytes, all of which are differentiation progeny of multipotent mesenchymal stromal cells (MSCs). However, also other stromal cells, fibroblasts, osteoclasts, and macrophages are part of the endosteal niche. On top, availability of ions, such as calcium, and the extracellular matrix, which is enriched with various growth factors, cytokines, adhesion molecules, and other signals, direct the properties and function of HSCs. Several studies have linked osteogenic cells, in particular osteoblasts, to HSCs, showing that HSCs home to endosteal niches, osteoblast numbers correlate with HSC numbers, and osteogenic cells produce factors, such as CXC chemokine ligand 12 (CXCL12), stem cell factor (SCF), granulocyte colony-stimulating factor (G-CSF), N-cadherin, angiopoietin-1, connexins, and Wnt and Notch signaling pathway components, which regulate HSC quiescence, proliferation, migration, and function [57, 58]. Many of the original findings were meanwhile largely confirmed with latest technologies [59–64]. These latter studies refined our understanding of the endosteal niche, suggesting that a) only few of the BM stromal cell subpopulations have potent HSC-supporting potential, b) the regulation of HSCs by osteogenic cells depends on their differentiation state with immature osteoblasts being more potent HSC supporters, c) HSC expansion depends on the bone turnover state and only takes place at bone remodeling-active sites, and d) homing of HSCs to endosteal niches is dynamically regulated and much different between steady-state and under stress conditions. These studies also collectively highlight that the endosteal niche is under constant reconstruction. In addition, as bone undergoes several changes during aging, all of these are also likely to affect the endosteal niche and HSCs.

Similar to HSCs, which undergo skewed differentiation into myeloid cells, MSCs favor adipogenic over osteogenic differentiation in aged humans and mice, leading to a shift from “red” to “yellow marrow” [65–67]. The resulting BM hypocellularity is characteristic for aged individuals. Adipocytes have been shown to impair B cell differentiation of HSCs and adipocyte numbers in aged individuals correlate with an increased density of maturing myeloid cells adjacent to adipocytes, suggesting that the age-related skewing of MSCs into the adipogenic lineage may contribute to myeloid skewing of HSCs [68, 69]. Adipocytes produce proinflammatory cytokines, which may facilitate the inflammatory microenvironment in aged BM and thereby exacerbate CH [70, 71]. Conversely, the reduced osteogenic differentiation of MSCs, which is associated with lower production of osteopontin (OPN), may negatively regulate HSC proliferation, and thus lead to accelerated HSC divisions and stem cell exhaustion during aging [72].

Besides the differentiation bias, other factors of MSCs may contribute to dysfunctional HSCs during aging, such as changes in the number and expression profiles of various MSC subsets and the reduced stemness properties that have been reported for aged MSCs [73–75]. A recent study more specifically addressed the role of stromal niche inflammation in age-related changes of the BM niche and HSCs [55]. The results identified endosteal MSCs as a relevant source of IL-1β in the inflamed and degraded aged BM niche, which may contribute to further inflammatory remodeling, chronic activation of emergency myelopoiesis pathways in HSCs, and impaired hematopoietic regeneration. Evidently, short-term IL-1 signaling blockade improved hematopoietic regeneration in old mice, while life-long IL-1R inhibition delayed niche aging and improved specific blood parameters [55]. Aging of MSCs also impacts on their capacity to adopt an immunosuppressive phenotype [76]. Moreover, the inflammatory environment in the BM is also key to increase receptor activator of NF-κB ligand (RANKL) expression by osteogenic cells, leading to enhanced bone degradation and thus, remodeling of the bone and BM niches [77].

An increased prevalence of senescent cells within the endosteal niche, including MSCs, has been documented in humans and mice and may also feed into the detrimental effects of chronic inflammation on the BM niche as described above [78, 79]. Besides the irreversible cell cycle arrest that senescent cells undergo, they also produce factors involved in matrix remodeling, pro-angiogenic factors, growth factors, and a wide variety of proinflammatory factors collectively termed senescence-associated secretory phenotype (SASP) [76]. A recent study in mice showed that by selectively depleting senescent MSCs or by systemic treatment with a senolytic drug, the metabolic fitness of HSCs, in particular in response to bacterial stimuli, could be improved [80]. Furthermore, increased senescence of MSCs has been observed in mice lacking Wnt signaling within the BM microenvironment, along with impaired endosteal niche and bone mass accrual, and age-dependent attenuated HSC repopulation activity and myeloid skewing [81]. Collectively, these data suggest that senescence of the endosteal niche may be amenable to therapeutic targeting and restoration of HSC functions during aging.

### The aging perivascular niche

The BM vasculature is composed of an intricate network of blood vessels, which supports bone homeostasis through the supply of oxygen, nutrients, and the secretion of angiocrine factors [82]. These blood vessels are formed by endothelial cells (ECs) exhibiting organ-specific transcriptional profiles, reflecting not only their ontogeny but also organ-specific function. ECs are a major source of pro-hematopoietic factors, such as Notch ligands (Jagged 1, Jagged 2, Delta-like ligands 1 and 4 (DLL1, DLL4)), E-selectin, angiogenin, as well as CXCL12 and SCF [63, 82]. Within the complex and multi-cellular skeletal system, ECs form vascular niches that have been reported to support both hematopoiesis and osteogenesis.

Vascular niches can be subdivided based on their location, vessel diameter, and expression of distinct marker sets. The arteriolar vasculature, characterized by high levels of cadherin 5 (CDH5) and stem cell antigen 1 (SCA1) expression, is directly connected to transitional type H vessels (CD31^high^, endomucin (EMCN)^high^), which then merge with sinusoidal type L vessels (CDH5^+^, CD31^low^, EMCN^low^). Type H vessels are located in the metaphysis in regions proximal to cortical bone in close association with osteoprogenitors and have been reported to support osteogenesis [83]. Type L vessels display a close network with leptin receptor (LEPR)-expressing perivascular stromal cells, which serve as progenitors to cells of the adipocyte lineage, and CXCL12-abundant reticular (CAR) cells, which support HSC function [84]. In this context, recent genetic lineage tracing and single-cell RNA sequencing identified specific LEPR^+^ subsets based on the co-expression of the osteogenic growth factor osteolectin: periarteriolar LEPR^+^ osteolectin^+^ cells primed for osteogenesis and perisinusoidal LEPR^+^ osteolectin^−^ cells primed for adipogenesis [61, 85]. Interestingly, LEPR^+^ stromal cells promote BM innervation by synthesizing nerve growth factor (NGF) and, in turn, nerve fibres promote hematopoietic and vascular regeneration by activating β-adrenergic receptor signaling in LEPR^+^ cells [86]. Thus, different vascular niches are associated with distinct perivascular cell types, forming cooperative niches that as an entity modulate HSC behavior.

Aging is associated with marked alterations in the BM vasculature as well as vascular niches [82]. Several studies have demonstrated an age-related reduction in arteries, arterioles, and type H vessels, which is in correlation with reduced osteogenesis, bone density, and endosteal niches [73, 83]. The sinusoidal vasculature contrarily seems to be less impacted and type L capillaries do not decline with age in mice [83]. Disrupted β-adrenergic sympathetic nerve signaling has been identified as an important determinant of niche remodeling during aging and may indirectly promote megakaryocyte differentiation through induction of stromal IL-6 [73]. These findings support the concept of an altered adrenergic-mesenchymal-hematopoietic axis directing lineage fate decisions in hematopoiesis during aging, specifically lymphopoiesis and megakaryopoiesis [87].

Concomitant with alterations in cellular composition, the aged vasculature displays a reduction in the expression of pro-hematopoietic factors, such as DLL4, netrin-1, SCF, and CXCL12, which are critical for HSC function [88–90]. In line, reconstitution of Notch ligands in ECs has been demonstrated to not only restore type H vessels but also perivascular niche function and HSC abundance [88, 91]. Loss of *Dll4* in ECs was further associated with increased myeloid skewing in mice [63]. Vascular aging has also been associated with elevated vessel leakiness, hypoxia, and elevated levels of reactive oxygen species (ROS) [90]. Additionally, chronic vascular inflammation has been shown to promote pre-mature HSC aging with reduced self-renewal and increased myeloid skewing [92, 93]. Together, these data support a critical role of the vasculature in the aging BM niche and a potential contribution to inflammaging and dysregulation of HSC function.

### Age-related immune cell changes

The BM functions also as a backbone of immunological memory, maintaining long-lived memory plasma cells and memory T cells, and hosts various other mature innate and adaptive immune cell types including B cells, regulatory T cells (Tregs), natural killer T (NKT) cells, monocytes, macrophages, dendritic cells, neutrophils, and myeloid-derived suppressor cells (MDSCs). Among these, lymphocytes represent a major fraction of total BM mononuclear cells, are distributed throughout stroma and parenchyma, and are condensed in lymphoid aggregates [54]. Altogether, immune cells provide a dynamic “immune niche” that influences HSCs during steady-state and emergency hematopoiesis directly by secretion of cytokines and/or indirectly by modulating MSCs [54]. With age, changes in the cellular composition and clonal involvement of immune niche cells may further contribute to CH.

Age-related changes of immune cells in humans have been mostly studied in peripheral blood [94–97], whereas much less is known about BM [98–101]. Reported changes in human BM include a more pronounced decline of naïve T cells compared to peripheral blood, a relative increase of effector/memory CD4^+^ T cells, and accumulation of highly activated CD8^+^ CD28^-^ T cells, while still maintaining a high number of polyfunctional memory CD4^+^ and CD8^+^ T cells [99]. Moreover, the BM is a significant reservoir for Tregs, which have both an immunosuppressive and tissue maintenance role and whose proportion has been shown to increase with age in mice [102]. This age-related accumulation of Tregs may contribute to reduced responsiveness of effector T cells. The proportions of human BM plasma and memory B cells within the CD19^+^ population were found to decrease [101]. This could impair protection against certain antigens in old age and may be a consequence of decreased CXCL12 expression in the aged, inflammatory BM microenvironment, leading to impaired homing of plasmablasts and diminished survival of plasma cells [101]. In contrast, inflammatory cytokine-producing plasma cells have been found to increase in numbers in the BM of old mice, where they stimulated myelopoiesis and regulated inflammatory gene expression by stromal cells, further contributing to inflammaging [56]. In terms of myeloid cells, BM macrophages in aged humans and mice were shown to exhibit an activated and inflammatory signature, which could contribute to induce a platelet bias in HSCs [103]. Senescent neutrophils, typically cleared by BM macrophages, increased in aged mice, consistent with functional macrophage defects [103]. Moreover, age-related secretion of grancalcin by macrophages has been demonstrated to induce skeletal stem/progenitor cell senescence in mice, thus impairing bone regeneration [104]. These studies exemplarily illustrate the instructive role of immune cell subsets in lineage skewing of HSCs and inflammatory niche alterations that occur with aging.

## The bi-directional impact of CH on the aging BM niche

The pre-malignant BM niche in CH carriers represents an opportunity to identify early changes before myeloid disease manifestation. Although direct experimental evidence linking CH to alterations of the BM microenvironment, and vice versa, is still limited, recent advances have been made. A central concept is that genetic subtypes of CHIP accelerate inflammaging of the BM niche, which progressively contributes to niche remodeling beyond normal aging (Fig. 1). Multiple studies have described enhanced inflammatory responses in *DNMT3A*-, *TET2*-, and *JAK2*-mutant myeloid cells [44]. In line with this, CHIP-carriers exhibited higher serum levels of IL-6, IL-8, TNF, IL-1β, and IL-18 compared to non-CHIP individuals [4, 13, 105]. In particular, driver gene-specific analyses showed an association of *TET2* with increased IL-1β, whereas *JAK2* and *SF3B1* were associated with increased circulating IL-18, highlighting the need for gene-specific analyses [4]. Importantly, mutant HSPCs have developed molecular mechanisms to adapt to these sustained inflammatory cues [44].

CHIP-related inflammatory signals appear to contribute to remodeling of the bone structure, in particular *DNMT3A*-mutant CHIP that has been linked to osteoporosis in humans [14]. Mechanistically, proinflammatory cytokines produced by *Dnmt3a*-deficient macrophages, including IL-20, enhanced osteoclastogenesis in mice, which demonstrated that CHIP progeny directly contributes to osteoporosis-inducing inflammation, osteoclast-mediated reduction in bone mass, and consequently endosteal niche deterioration [14]. Hematopoietic-specific inactivation of *Tet2* in mice also resulted in reduced bone mass but the effects on bone phenotype were milder [14]. It is important to note that several growth factors/proteins released during and/or regulating bone resorption, such as TGF-β and bone morphogenetic proteins (BMPs), have been implicated in the progression of MDS [106, 107].

With regard to MSCs, more conclusive data are awaited from ongoing studies investigating the differentiation capacity and HSC supportive function of MSCs obtained from CHIP carriers. An influence of sympathetic neuropathy, driven by mutant cells, on MSCs is conceivable, which could compromise their HSC-supportive function and contribute to clonal expansion [108, 109]. Further, *Dnmt3a*-mutant HSPCs were shown to induce senescence of MSCs through the production of IL-6 [110], which in turn may contribute to CH and myeloid skewing as discussed earlier.

CHIP progeny may also drive remodeling of BM vascular niches to form a favorable niche that supports clonal expansion. Recent findings in a lung cancer model suggested that *Tet2*-deficient immune cells, specifically myeloid cells, promote angiogenesis, possibly through enhanced S100A8/A9 secretion [111]. Therefore, the inflammatory signatures that are attributable to defined CHIP progeny could increase vascularization and perivascular permeability, as well as further promote the activation of inflammatory transcriptional programs in aged BM stromal and endothelial cells [112–114].

Paracrine inflammatory signals from the aging and adipocyte-enriched BM microenvironment may, in turn, confer a selective advantage to *DNMT3A*- or *TET2*-mutant CHIP clones [70, 71]. Aging-related prolonged TNF signaling in an inflammatory environment has been shown to favor *Dnmt3a*^R878H^-mutant (human: *DNMT3A*^R882H^) and *Tet2*-knockout CH [115–117]. Further, IL-1R signaling has been recently implicated as a driver of *Tet2*^+/−^ CHIP progression during aging [118]. In addition, driver mutations in *TET2* may contribute to repress NK cell-mediated immune surveillance of malignant clones in the BM [119]. Together, these studies point out a continuous bi-directional inflammatory program in the aging BM niche that supports and is further driven by mutant HSPCs and their progeny, leading to remodeling of the endosteal niche while transforming the vascular niche to a selective microenvironment that favors mutated clones. This combined intrinsic and extrinsic fitness advantage and subsequent clonal expansion are the defining features of CH, which, when exacerbated, increases the likelihood of transformation to MDS and associated hematologic malignancies.

## Embracing complexity: new tools to study the CH BM niche

In recent years, single-cell-sequencing approaches have revolutionized our understanding of normal and malignant hematopoiesis by enabling the precise characterization of the transitional cell states between hematopoietic hierarchy stages and their HSPC onset clonal origins that are dysregulated during hematologic malignancies [120, 121]. Knowing the characteristics of normal cells should facilitate understanding their malignant counterparts and thus provide clues on how CH shifts the HSPC transcriptional landscape toward clonal expansion and lineage skewing. A recent breakthrough was achieved using a single-cell multi-omics approach (combining scRNA-seq, chromatin accessibility, methylome), showing that *Tet2* and *Dnmt3a* loss differentially affect DNA-binding motifs of key cell fate transcription factors, causing opposite shifts in the frequencies of erythroid versus myelomonocytic progenitors [122]. Single-cell RNA-seq approaches allow to directly compare mutated and wild-type cell transcriptomics within the same individual, and have also been applied to resolve the BM niche cellular heterogeneity, pinpoint the major pro-hematopoietic factors, and characterize their secreting supportive cells [27, 31, 63, 64, 123–125]. This has refined our current knowledge of the BM niche architecture and its influence on inducing HSPCs toward distinct lineage commitment paths. Interestingly, monocytic production appears to be spatially separated from other lineages and instructed by a small subpopulation of endothelial cells producing macrophage colony-stimulating factor (M-CSF/CSF-1), although whether such functional output is regulated directly by M-CSF/CSF-1 remains to be elucidated [126]. Such spatial organization has also been identified for other stromal cells using spatial transcriptomics. Specifically, two CAR cell subsets differentially localized to sinusoidal or arteriolar surfaces, acting as “professional” cytokine-secreting stromal cells to establish distinct perivascular micro-niches to support HSPCs in mice [61]. Recent efforts using the same spatial approach showed early hints toward stromal cell subsets with specific cytokine secretion in human fetal hematopoiesis [127, 128].

**Fig. 2:**
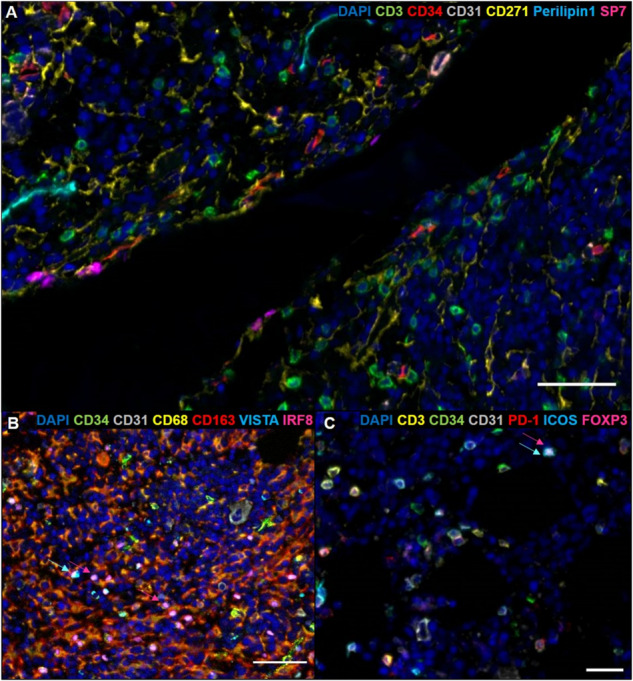
Multi-parameter fluorescence immunohistochemistry of human BM sections. Multiplex TSA-based staining of 3 formalin-fixed, paraffin-embedded BM tissue slides from a 72-year old, healthy donor was performed with a seven-color multiplex immunofluorescence protocol for six marker molecules plus nuclei (DAPI, dark blue) and analyzed by multispectral imaging with the Vectra 3.0 imaging platform. **A** Representative staining of cells with antibodies directed against CD3 (T cells, green), CD34 (HSCs and endothelial cells, red), CD31 (endothelial cells, gray), CD271 (MSCs, yellow), SP7 (osteoblasts, magenta), and perilipin 1 (adipocytes, cyan) is shown. Scale bare, 100 µm. **B** BM macrophages were characterized by utilizing antibodies against CD68 (macrophages, yellow), CD163 (macrophages, red), IRF8 (transcription factor, magenta), VISTA (immunosuppressive receptor, cyan), CD34 (HSCs and endothelial cells, green), and CD31 (endothelial cells, gray). Arrows highlight a representative example of VISTA^+^ (cyan arrow) and IRF8^+^ (magenta arrow) cells. Scale bare, 50 µm. **C** T cells were determined using antibodies against CD3 (T cells, yellow), PD-1 (immune inhibitory receptor, red), FOXP3 (transcription factor of Tregs, magenta), ICOS (activating receptor, cyan), CD34 (HSCs and endothelial cells, green), and CD31 (endothelial cells, gray). Arrows mark a representative example of an ICOS^+^ (cyan arrow) and FOXP3^+^ (magenta arrow) cell. Scale bare, 50 µm. Original magnification was x200 in all slides.

Comprehensive single-cell data sets for BM across healthy donors and over donor lifespan are being generated already and can serve as valuable ref [129, 130]. In future studies, a better characterization of the BM niche in human adult and aging hematopoiesis will be essential to close the gap between the anatomical and cellular difference of stromal cell identities and their supportive roles compared to those identified in mouse models. More importantly, it would be of great interest to explore if CH cells utilize a different spatial BM niche organization that dysbalances hematopoietic differentiation, and investigate their interaction with different cell populations of the hematopoietic and immune system, as well as vessels, fat, bone, and connective tissue structures. From a practical point of view, BM tissues can develop a high degree of autofluorescence and fragility due to fixation and decalcification procedures, which often vary across different laboratories [131]. Therefore, BM tissue poses a challenge for multi-parameter fluorescence immunohistochemistry protocols. Nevertheless, several technologies are available to overcome these challenges, such as spatial profiling of proteins or RNA via GeoMx® Digital Spatial Profiler (NanoString) [132], epitope-targeted mass spectrometry in the Hyperion™ Imaging System (Standard BioTools) [133], tyramide signal amplification (TSA) visualized by PhenoImagers (formerly Vectra® Polaris™, Akoya Biosciences) [134], and cyclic immunofluorescence staining (ICS) with the PhenoCycler™ (formerly CODEX®, Akoya Biosciences) [135] or the MACSima™ imaging platform (Miltenyi Biotec) [136]. Based on our experience with the TSA method using the Vectra 3.0 spectral imaging system and the ICS method using the MACSima imaging platform, both methods are suitable to obtain robust results on formalin-fixed, paraffin-embedded BM sections. The TSA method allows for whole-tissue scanning, high sensitivity to detect weakly expressed markers, and the acquisition of multispectral images resolving up to seven marker molecules, including the DNA stain DAPI, on a single tissue section (Fig. 2). The obtained high-resolution data are useful for functional characterization and distance measurements between cells of interest. However, detailed analysis of several cell populations is not possible in one run and requires the use of multiple staining protocols on separate tissues. Other developments like the fully automated system of the MACSima imaging platform offer the possibility of analyzing >100 markers in order to determine phenotypic and functional properties of different cell populations. This ICS-based procedure relies on iterative staining with up to three different commercially available fluorophore-conjugated antibodies and DAPI per cycle. The resulting stack of marker images could provide unprecedented insight into the spatial distribution and interactions of different cell populations in the BM niche.

## Clinical management of individuals with CH

Increasing accessibility of NGS assays has led to a growing population of individuals who are aware that they are CH carriers. Since CH is ubiquitous in individuals >70 years and the overall risk for transformation into a hematopoietic neoplasm is small, it will be crucial to establish a comprehensive prognostic model for transformation risk in the future. A group from Dana-Farber Cancer Institute recently presented a CH risk score (CHRS) established retrospectively from a cohort of 193743 healthy UK Biobank participants and validated in two independent CHIP/CCUS cohorts [137]. Prognostically unfavorable determinants of progression to hematologic neoplasm included genetic features (high-risk mutations, >1 mutation, and VAF ≥ 0.2), patient age (≥65 years), the presence of cytopenia (CCUS versus CHIP), and high red blood cell indices [137]. Another recent study presented a different approach that calculates the likelihood of developing different types of myeloid neoplasms over 15 years and is accessible via a web-based application [138]. In the future, it will be necessary to validate these retrospective data from the UK Biobank in prospective studies conducted in dedicated CHIP clinics, as have been established at many academic centers, including ours. Further, CH associates with several other non-hematological disease states and inflammation-associated comorbidities. Hence, CH carriers should be evaluated for the presence of CVD, osteoporosis, osteoarthritis, and autoimmune disorders, etc., especially in the presence of additional risk factors. Taken together, these observations underscore the importance of adequately monitoring individuals with CH and high-risk features as well as the need for evidence-based clinical guidelines. The latter should also involve safe and effective interventions currently explored in early phase studies. Importantly, close collaboration with specialists in non-hematologic sequelae of CH will be crucial in this emerging topic in personalized medicine.

## Future directions

Clearly, a progressively disturbed microenvironment with impaired function of supportive niche and immune cells plays a major role in the dysregulation of normal hematopoiesis and progression to hematologic malignancy. A better understanding of the niche alterations in the premalignant state of CH could expose levers for preventive treatments in higher-risk CH, apart from mutation-specific targeted therapies [139]. Studies of serial samples may also provide insights into niche contributions to varying trajectories of a clone between individuals.

Moving on from the current detection of CH in bulk populations, an important goal in the field is to resolve the cellular distribution of clonal mutations and their dynamics. CH progeny may have an intrinsically different biology and broadly impact the BM microenvironment as well as other tissues and host immune function. Growing evidence supports a role for CHIP in inflammaging, thereby potentiating age-related inflammatory changes in the BM and other tissues. The identification of common and mutation-specific alterations in the BM niche of CH carriers, perhaps focusing on those with high-risk features or larger clone size, may help to find features that could be targeted to suppress the self-reinforcing pre-leukemic BM microenvironment, dislodge mutant clones from their protective niche, and restore healthy/balanced hematopoiesis. To address this will require ambitious translational studies that assess single-cell transcriptomics of mutant and wild-type cells and resolve the spatial BM niche organization in human specimens. Biopsies may be obtained from leftover material from routine surgical intervention (e.g. hip replacements) or during diagnostic work-up of individuals with suspected hematologic disorder. Importantly, results from such studies will contribute to build a comprehensive atlas of the intact and pre-malignant aging human BM niche, and advance our knowledge of the aging process and the contribution of CH.

## Data Availability

No data to deposit in a repository; further data are available upon request from the corresponding authors.

## References

[1] Dawoud AAZ, Tapper WJ, Cross NCP (2020). Clonal myelopoiesis in the UK Biobank cohort: ASXL1 mutations are strongly associated with smoking. Leukemia.

[2] De-Morgan A, Meggendorfer M, Haferlach C, Shlush L (2021). Male predominance in AML is associated with specific preleukemic mutations. Leukemia.

[3] Kar SP, Quiros PM, Gu M, Jiang T, Mitchell J, Langdon R (2022). Genome-wide analyses of 200,453 individuals yield new insights into the causes and consequences of clonal hematopoiesis. Nat Genet.

[4] Bick AG, Weinstock JS, Nandakumar SK, Fulco CP, Bao EL, Zekavat SM (2020). Inherited causes of clonal haematopoiesis in 97,691 whole genomes. Nature.

[5] Steensma DP, Bejar R, Jaiswal S, Lindsley RC, Sekeres MA, Hasserjian RP (2015). Clonal hematopoiesis of indeterminate potential and its distinction from myelodysplastic syndromes. Blood.

[6] Jaiswal S, Fontanillas P, Flannick J, Manning A, Grauman PV, Mar BG (2014). Age-Related Clonal Hematopoiesis Associated with Adverse Outcomes. N Engl J Med.

[7] Genovese G, Kähler AK, Handsaker RE, Lindberg J, Rose SA, Bakhoum SF (2014). Clonal Hematopoiesis and Blood-Cancer Risk Inferred from Blood DNA Sequence. N Engl J Med.

[8] Cargo CA, Rowbotham N, Evans PA, Barrans SL, Bowen DT, Crouch S (2015). Targeted sequencing identifies patients with preclinical MDS at high risk of disease progression. Blood.

[9] Abelson S, Collord G, Ng SWK, Weissbrod O, Mendelson Cohen N, Niemeyer E (2018). Prediction of acute myeloid leukaemia risk in healthy individuals. Nature.

[10] Desai P, Mencia-Trinchant N, Savenkov O, Simon MS, Cheang G, Lee S (2018). Somatic mutations precede acute myeloid leukemia years before diagnosis. Nat Med.

[11] Takahashi K, Wang F, Kantarjian H, Doss D, Khanna K, Thompson E (2017). Preleukaemic clonal haemopoiesis and risk of therapy-related myeloid neoplasms: a case-control study. Lancet Oncol.

[12] Coombs CC, Zehir A, Devlin SM, Kishtagari A, Syed A, Jonsson P (2017). Therapy-Related Clonal Hematopoiesis in Patients with Non-hematologic Cancers Is Common and Associated with Adverse Clinical Outcomes. Cell Stem Cell.

[13] Jaiswal S, Natarajan P, Silver AJ, Gibson CJ, Bick AG, Shvartz E (2017). Clonal Hematopoiesis and Risk of Atherosclerotic Cardiovascular Disease. N Engl J Med.

[14] Kim PG, Niroula A, Shkolnik V, McConkey M, Lin A, Słabicki M (2021). Dnmt3a-mutated clonal hematopoiesis promotes osteoporosis. J Exp Med.

[15] Agrawal M, Niroula A, Cunin P, McConkey M, Shkolnik V, Kim PG (2022). TET2-mutant clonal hematopoiesis and risk of gout. Blood.

[16] Wong WJ, Emdin C, Bick AG, Zekavat SM, Niroula A, Pirruccello JP (2023). Clonal haematopoiesis and risk of chronic liver disease. Nature.

[17] Hecker JS, Hartmann L, Rivière J, Buck MC, van der Garde M, Rothenberg-Thurley M (2021). CHIP and hips: clonal hematopoiesis is common in patients undergoing hip arthroplasty and is associated with autoimmune disease. Blood.

[18] Ayachi S, Buscarlet M, Busque L (2020). 60 Years of clonal hematopoiesis research: From X-chromosome inactivation studies to the identification of driver mutations. Exp Hematol.

[19] Bouzid H, Belk JA, Jan M, Qi Y, Sarnowski C, Wirth S (2023). Clonal hematopoiesis is associated with protection from Alzheimer’s disease. Nat Med.

[20] Khoury JD, Solary E, Abla O, Akkari Y, Alaggio R, Apperley JF (2022). The 5th edition of the World Health Organization Classification of Haematolymphoid Tumours: Myeloid and Histiocytic/Dendritic Neoplasms. Leukemia.

[21] Hartmann L, Hecker JS, Rothenberg-Thurley M, Rivière J, Jentzsch M, Ksienzyk B (2022). Compartment-specific mutational landscape of clonal hematopoiesis. Leukemia.

[22] Niroula A, Sekar A, Murakami MA, Trinder M, Agrawal M, Wong WJ (2021). Distinction of lymphoid and myeloid clonal hematopoiesis. Nat Med.

[23] Mitchell E, Spencer Chapman M, Williams N, Dawson KJ, Mende N, Calderbank EF (2022). Clonal dynamics of haematopoiesis across the human lifespan. Nature.

[24] Fabre MA, de Almeida JG, Fiorillo E, Mitchell E, Damaskou A, Rak J (2022). The longitudinal dynamics and natural history of clonal haematopoiesis. Nature.

[25] Baldow C, Thielecke L, Glauche I (2016). Model based analysis of clonal developments allows for early detection of monoclonal conversion and leukemia. PLoS One.

[26] Vedi A, Hayler D, Biezuner T, Santoro A, Sham K, Tuval A (2021). DNMT3A R882 Mutation in Human Haematopoietic Stem Cells Alters Differentiation Towards Neutrophils and Monocytes. Blood.

[27] Nam AS, Dusaj N, Izzo F, Murali R, Myers RM, Mouhieddine TH (2022). Single-cell multi-omics of human clonal hematopoiesis reveals that DNMT3A R882 mutations perturb early progenitor states through selective hypomethylation. Nat Genet.

[28] Huerga Encabo H, Aramburu IV, Garcia-Albornoz M, Piganeau M, Wood H, Song A (2023). Loss of TET2 in human hematopoietic stem cells alters the development and function of neutrophils. Cell Stem Cell.

[29] Arends CM, Galan-Sousa J, Hoyer K, Chan W, Jäger M, Yoshida K (2018). Hematopoietic lineage distribution and evolutionary dynamics of clonal hematopoiesis. Leukemia.

[30] Buscarlet M, Provost S, Zada YF, Bourgoin V, Mollica L, Dubé MP (2018). Lineage restriction analyses in CHIP indicate myeloid bias for TET2 and multipotent stem cell origin for DNMT3A. Blood.

[31] Abplanalp WT, Schuhmacher B, Cremer S, Merten M, Shumliakivska M, Macinkovic I (2023). Cell-intrinsic effects of clonal hematopoiesis in heart failure. Nat Cardiovasc Res.

[32] Watson CJ, Papula AL, Poon GYP, Wong WH, Young AL, Druley TE (2020). The evolutionary dynamics and fitness landscape of clonal hematopoiesis. Science.

[33] Robertson NA, Latorre-Crespo E, Terradas-Terradas M, Lemos-Portela J, Purcell AC, Livesey BJ (2022). Longitudinal dynamics of clonal hematopoiesis identifies gene-specific fitness effects. Nat Med.

[34] Williams N, Lee J, Mitchell E, Moore L, Baxter EJ, Hewinson J (2022). Life histories of myeloproliferative neoplasms inferred from phylogenies. Nature.

[35] Buck MC, Bast L, Hecker JS, Rivière J, Rothenberg-Thurley M, Vogel L (2023). Progressive disruption of hematopoietic architecture from clonal hematopoiesis to MDS. iScience.

[36] Jakobsen NA, Turkalj S, Zeng AGX, Stoilova B, Metzner M, Nagree MS et al. Selective advantage of mutant stem cells in clonal hematopoiesis occurs by attenuating the deleterious effects of inflammation and aging. bioRxiv. 2023. https://www.biorxiv.org/content/10.1101/2023.09.12.557322v1.

[37] Zeng AGX, Nagree MS, Jakobsen NA, Shah S, Murison A, Cheong J-G et al. A hematopoietic stem cell subset that retains memory of prior inflammatory stress accumulates in aging and clonal hematopoiesis. bioRxiv. 2023. https://www.biorxiv.org/content/10.1101/2023.09.11.557271v1.

[38] Osman AEG, Mencia-Trinchant N, Saygin C, Moma L, Kim A, Housman G (2023). Paired bone marrow and peripheral blood samples demonstrate lack of widespread dissemination of some CH clones. Blood Adv.

[39] Nowakowska MK, Kim TT, Thompson MT, Bolton KL, Deswal A, Lin SH (2022). Association of clonal hematopoiesis mutations with clinical outcomes: A systematic review and meta-analysis. Am J Hematol.

[40] Stein A, Metzeler K, Kubasch AS, Rommel KP, Desch S, Buettner P (2022). Clonal hematopoiesis and cardiovascular disease: deciphering interconnections. Basic Res Cardiol.

[41] Heyde A, Rohde D, McAlpine CS, Zhang S, Hoyer FF, Gerold JM (2021). Increased stem cell proliferation in atherosclerosis accelerates clonal hematopoiesis. Cell.

[42] Kessler MD, Damask A, O’Keeffe S, Banerjee N, Li D, Watanabe K (2022). Common and rare variant associations with clonal haematopoiesis phenotypes. Nature.

[43] Gao T, Ptashkin R, Bolton KL, Sirenko M, Fong C, Spitzer B (2021). Interplay between chromosomal alterations and gene mutations shapes the evolutionary trajectory of clonal hematopoiesis. Nat Commun.

[44] Caiado F, Pietras EM, Manz MG (2021). Inflammation as a regulator of hematopoietic stem cell function in disease, aging, and clonal selection. J Exp Med..

[45] Bolton KL, Koh Y, Foote MB, Im H, Jee J, Sun CH (2021). Clonal hematopoiesis is associated with risk of severe Covid-19. Nat Commun.

[46] Zhou Y, Shalhoub R, Rogers SN, Yu S, Gu M, Fabre MA (2022). Clonal hematopoiesis is not significantly associated with COVID-19 disease severity. Blood.

[47] Zekavat SM, Lin SH, Bick AG, Liu A, Paruchuri K, Wang C (2021). Hematopoietic mosaic chromosomal alterations increase the risk for diverse types of infection. Nat Med.

[48] Datzmann T, Trautmann F, Tesch F, Mies A, Hofbauer LC, Platzbecker U (2018). Associations of myeloid hematological diseases of the elderly with osteoporosis: A longitudinal analysis of routine health care data. Leuk Res.

[49] Weidner H, Rauner M, Trautmann F, Schmitt J, Balaian E, Mies A (2017). Myelodysplastic syndromes and bone loss in mice and men. Leukemia.

[50] Sano S, Oshima K, Wang Y, MacLauchlan S, Katanasaka Y, Sano M (2018). Tet2-Mediated Clonal Hematopoiesis Accelerates Heart Failure Through a Mechanism Involving the IL-1β/NLRP3 Inflammasome. J Am Coll Cardiol.

[51] Ridker PM, Everett BM, Thuren T, MacFadyen JG, Chang WH, Ballantyne C (2017). Antiinflammatory Therapy with Canakinumab for Atherosclerotic Disease. N Engl J Med.

[52] Schieker M, Conaghan PG, Mindeholm L, Praestgaard J, Solomon DH, Scotti C (2020). Effects of interleukin-1β inhibition on incident hip and knee replacement: Exploratory analyses from a randomized, double-blind, placebo-controlled trial. Ann Intern Med.

[53] Pinho S, Frenette PS (2019). Haematopoietic stem cell activity and interactions with the niche. Nat Rev Mol Cell Biol.

[54] Riether C, Schürch CM, Ochsenbein AF (2015). Regulation of hematopoietic and leukemic stem cells by the immune system. Cell Death Differ.

[55] Mitchell CA, Verovskaya EV, Calero-Nieto FJ, Olson OC, Swann JW, Wang X (2023). Stromal niche inflammation mediated by IL-1 signalling is a targetable driver of haematopoietic ageing. Nat Cell Biol.

[56] Pioli PD, Casero D, Montecino-Rodriguez E, Morrison SL, Dorshkind K (2019). Plasma Cells Are Obligate Effectors of Enhanced Myelopoiesis in Aging Bone Marrow. Immunity.

[57] Kim MJ, Valderrábano RJ, Wu JY (2022). Osteoblast Lineage Support of Hematopoiesis in Health and Disease. J Bone Min Res.

[58] Zhao M, Tao F, Venkatraman A, Li Z, Smith SE, Unruh J (2019). N-Cadherin-Expressing Bone and Marrow Stromal Progenitor Cells Maintain Reserve Hematopoietic Stem Cells. Cell Rep.

[59] Kokkaliaris KD, Kunz L, Cabezas-Wallscheid N, Christodoulou C, Renders S, Camargo F (2020). Adult blood stem cell localization reflects the abundance of reported bone marrow niche cell types and their combinations. Blood.

[60] Christodoulou C, Spencer JA, Yeh SCA, Turcotte R, Kokkaliaris KD, Panero R (2020). Live-animal imaging of native haematopoietic stem and progenitor cells. Nature.

[61] Baccin C, Al-Sabah J, Velten L, Helbling PM, Grünschläger F, Hernández-Malmierca P (2020). Combined single-cell and spatial transcriptomics reveal the molecular, cellular and spatial bone marrow niche organization. Nat Cell Biol.

[62] Severe N, Karabacak NM, Gustafsson K, Baryawno N, Courties G, Kfoury Y (2019). Stress-Induced Changes in Bone Marrow Stromal Cell Populations Revealed through Single-Cell Protein Expression Mapping. Cell Stem Cell.

[63] Tikhonova AN, Dolgalev I, Hu H, Sivaraj KK, Hoxha E, Cuesta-Domínguez Á (2019). The bone marrow microenvironment at single-cell resolution. Nature.

[64] Baryawno N, Przybylski D, Kowalczyk MS, Kfoury Y, Severe N, Gustafsson K (2019). A Cellular Taxonomy of the Bone Marrow Stroma in Homeostasis and Leukemia. Cell.

[65] Alameda D, Saez B, Lara-Astiaso D, Sarvide S, Lasa M, Alignani D (2020). Characterization of freshly isolated bone marrow mesenchymal stromal cells from healthy donors and patients with multiple myeloma: transcriptional modulation of the microenvironment. Haematologica.

[66] Moerman EJ, Teng K, Lipschitz DA, Lecka-Czernik B (2004). Aging activates adipogenic and suppresses osteogenic programs in mesenchymal marrow stroma/stem cells: the role of PPAR-gamma2 transcription factor and TGF-beta/BMP signaling pathways. Aging Cell.

[67] Wu M, Wang Y, Shao JZ, Wang J, Chen W, Li YP (2017). Cbfβ governs osteoblast−adipocyte lineage commitment through enhancing β-catenin signaling and suppressing adipogenesis gene expression. Proc Natl Acad Sci USA.

[68] Kennedy DE, Knight KL (2015). Inhibition of B Lymphopoiesis by Adipocytes and IL-1-Producing Myeloid-Derived Suppressor Cells. J Immunol.

[69] Aguilar-Navarro AG, Meza-León B, Gratzinger D, Juárez-Aguilar FG, Chang Q, Ornatsky O (2020). Human Aging Alters the Spatial Organization between CD34+ Hematopoietic Cells and Adipocytes in Bone Marrow. Stem Cell Rep.

[70] Pasupuleti SK, Ramdas B, Burns SS, Palam LR, Kanumuri R, Kumar R (2023). Obesity-induced inflammation exacerbates clonal hematopoiesis. J Clin Invest.

[71] Zioni N, Bercovich AA, Chapal-Ilani N, Bacharach T, Rappoport N, Solomon A (2023). Inflammatory signals from fatty bone marrow support DNMT3A driven clonal hematopoiesis. Nat Commun.

[72] Guidi N, Sacma M, Ständker L, Soller K, Marka G, Eiwen K (2017). Osteopontin attenuates aging-associated phenotypes of hematopoietic stem cells. EMBO J.

[73] Ho YH, del Toro R, Rivera-Torres J, Rak J, Korn C, García-García A (2019). Remodeling of Bone Marrow Hematopoietic Stem Cell Niches Promotes Myeloid Cell Expansion during Premature or Physiological Aging. Cell Stem Cell.

[74] Saçma M, Pospiech J, Bogeska R, de Back W, Mallm JP, Sakk V (2019). Haematopoietic stem cells in perisinusoidal niches are protected from ageing. Nat Cell Biol.

[75] Maryanovich M, Zahalka AH, Pierce H, Pinho S, Nakahara F, Asada N (2018). Adrenergic nerve degeneration in bone marrow drives aging of the hematopoietic stem cell niche. Nat Med.

[76] Massaro F, Corrillon F, Stamatopoulos B, Meuleman N, Lagneaux L, Bron D (2020). Aging of Bone Marrow Mesenchymal Stromal Cells: Hematopoiesis Disturbances and Potential Role in the Development of Hematologic Cancers. Cancers.

[77] Lin TH, Gibon E, Loi F, Pajarinen J, Córdova LA, Nabeshima A (2017). Decreased osteogenesis in mesenchymal stem cells derived from the aged mouse is associated with enhanced NF-κB activity. J Orthop Res.

[78] Zhang DY, Wang HJ, Tan YZ (2011). Wnt/β-Catenin Signaling Induces the Aging of Mesenchymal Stem Cells through the DNA Damage Response and the p53/p21 Pathway. PLoS One.

[79] Zhou S, Greenberger JS, Epperly MW, Goff JP, Adler C, Leboff MS (2008). Age-related intrinsic changes in human bone-marrow-derived mesenchymal stem cells and their differentiation to osteoblasts. Aging Cell.

[80] Hellmich C, Wojtowicz E, Moore JA, Mistry JJ, Jibril A, Johnson BB (2023). p16INK4A-dependent senescence in the bone marrow niche drives age-related metabolic changes of hematopoietic progenitors. Blood Adv.

[81] Poudel SB, So HS, Sim HJ, Cho JS, Cho ES, Jeon YM (2021). Osteoblastic Wntless deletion differentially regulates the fate and functions of bone marrow-derived stem cells in relation to age. Stem Cells.

[82] Owen-Woods C, Kusumbe A (2022). Fundamentals of bone vasculature: Specialization, interactions and functions. Semin Cell Dev Biol.

[83] Kusumbe AP, Ramasamy SK, Adams RH (2014). Coupling of angiogenesis and osteogenesis by a specific vessel subtype in bone. Nature.

[84] Acar M, Kocherlakota KS, Murphy MM, Peyer JG, Oguro H, Inra CN (2015). Deep imaging of bone marrow shows non-dividing stem cells are mainly perisinusoidal. Nature.

[85] Shen B, Tasdogan A, Ubellacker JM, Zhang J, Nosyreva ED, Du L (2021). A mechanosensitive peri-arteriolar niche for osteogenesis and lymphopoiesis. Nature.

[86] Gao X, Murphy MM, Peyer JG, Ni Y, Yang M, Zhang Y (2023). Leptin receptor+ cells promote bone marrow innervation and regeneration by synthesizing nerve growth factor. Nat Cell Biol.

[87] Raaijmakers MHGPGP (2019). Aging of the Hematopoietic Stem Cell Niche: An Unnerving Matter. Cell Stem Cell.

[88] Ramasamy SK, Kusumbe AP, Schiller M, Zeuschner D, Bixel MG, Milia C (2016). Blood flow controls bone vascular function and osteogenesis. Nat Commun.

[89] Renders S, Svendsen AF, Panten J, Rama N, Maryanovich M, Sommerkamp P (2021). Niche derived netrin-1 regulates hematopoietic stem cell dormancy via its receptor neogenin-1. Nat Commun.

[90] Poulos MG, Ramalingam P, Gutkin MC, Llanos P, Gilleran K, Rabbany SY (2017). Endothelial transplantation rejuvenates aged hematopoietic stem cell function. J Clin Invest.

[91] Kusumbe AP, Ramasamy SK, Itkin T, Mäe MA, Langen UH, Betsholtz C (2016). Age-dependent modulation of vascular niches for haematopoietic stem cells. Nature.

[92] Ramalingam P, Poulos MG, Lazzari E, Gutkin MC, Lopez D, Kloss CC (2020). Chronic activation of endothelial MAPK disrupts hematopoiesis via NFKB dependent inflammatory stress reversible by SCGF. Nat Commun.

[93] Pietras EM, Mirantes-Barbeito C, Fong S, Loeffler D, Kovtonyuk LV, Zhang S (2016). Chronic interleukin-1 exposure drives haematopoietic stem cells towards precocious myeloid differentiation at the expense of self-renewal. Nat Cell Biol.

[94] Huang Z, Chen B, Liu X, Li H, Xie L, Gao Y (2021). Effects of sex and aging on the immune cell landscape as assessed by single-cell transcriptomic analysis. Proc Natl Acad Sci USA.

[95] Mogilenko DA, Shpynov O, Andhey PS, Arthur L, Swain A, Esaulova E (2021). Comprehensive Profiling of an Aging Immune System Reveals Clonal GZMK+ CD8+ T Cells as Conserved Hallmark of Inflammaging. Immunity.

[96] Hashimoto K, Kouno T, Ikawa T, Hayatsu N, Miyajima Y, Yabukami H (2019). Single-cell transcriptomics reveals expansion of cytotoxic CD4 T cells in supercentenarians. Proc Natl Acad Sci USA.

[97] Mogilenko DA, Shchukina I, Artyomov MN (2022). Immune ageing at single-cell resolution. Nat Rev Immunol.

[98] Hennrich ML, Romanov N, Horn P, Jaeger S, Eckstein V, Steeples V (2018). Cell-specific proteome analyses of human bone marrow reveal molecular features of age-dependent functional decline. Nat Commun.

[99] Herndler-Brandstetter D, Landgraf K, Tzankov A, Jenewein B, Brunauer R, Laschober GT (2012). The impact of aging on memory T cell phenotype and function in the human bone marrow. J Leukoc Biol.

[100] Okhrimenko A, Grün JR, Westendorf K, Fang Z, Reinke S, Von Roth P (2014). Human memory T cells from the bone marrow are resting and maintain long-lasting systemic memory. Proc Natl Acad Sci USA.

[101] Pritz T, Lair J, Ban M, Keller M, Weinberger B, Krismer M (2015). Plasma cell numbers decrease in bone marrow of old patients. Eur J Immunol.

[102] Fischer L, Herkner C, Kitte R, Dohnke S, Riewaldt J, Kretschmer K (2019). Foxp3+ Regulatory T Cells in Bone and Hematopoietic Homeostasis. Front Endocrinol.

[103] Frisch BJ, Hoffman CM, Latchney SE, LaMere MW, Myers J, Ashton J (2019). Aged marrow macrophages expand platelet-biased hematopoietic stem cells via interleukin-1B. JCI Insight.

[104] Zou NY, Liu R, Huang M, Jiao YR, Wei J, Jiang Y (2024). Age-related secretion of grancalcin by macrophages induces skeletal stem/progenitor cell senescence during fracture healing. Bone Res.

[105] Cook EK, Izukawa T, Young S, Rosen G, Jamali M, Zhang L (2019). Comorbid and inflammatory characteristics of genetic subtypes of clonal hematopoiesis. Blood Adv.

[106] Zhou L, McMahon C, Bhagat T, Alencar C, Yu Y, Fazzari M (2011). Reduced SMAD7 leads to overactivation of TGF-β signaling in MDS that can be reversed by a specific inhibitor of TGF-β receptor I kinase. Cancer Res.

[107] Fenaux P, Kiladjian JJ, Platzbecker U (2019). Luspatercept for the treatment of anemia in myelodysplastic syndromes and primary myelofibrosis. Blood.

[108] Arranz L, Sánchez-Aguilera A, Martín-Pérez D, Isern J, Langa X, Tzankov A (2014). Neuropathy of haematopoietic stem cell niche is essential for myeloproliferative neoplasms. Nature.

[109] Kovtun I, von Bonin M, Ibneeva L, Frimmel J, Middeke JM, Kunadt D (2023). Profound sympathetic neuropathy in the bone marrow of patients with acute myeloid leukemia. Leukemia.

[110] Mistry JJ, Young KA, Trowbridge JJ (2022). Bone Marrow Stromal Cell Senescence Induced By Dnmt3a-Mutant Hematopoietic Stem and Progenitor Cells Accelerates Clonal Hematopoiesis and Progression to Leukemia. Blood.

[111] Nguyen YTM, Fujisawa M, Nguyen TB, Suehara Y, Sakamoto T, Matsuoka R (2021). Tet2 deficiency in immune cells exacerbates tumor progression by increasing angiogenesis in a lung cancer model. Cancer Sci.

[112] Fuster JJ, MacLauchlan S, Zuriaga MA, Polackal MN, Ostriker AC, Chakraborty R (2017). Clonal hematopoiesis associated with TET2 deficiency accelerates atherosclerosis development in mice. Science.

[113] Abplanalp WT, Cremer S, John D, Hoffmann J, Schuhmacher B, Merten M (2021). Clonal Hematopoiesis-Driver DNMT3A Mutations Alter Immune Cells in Heart Failure. Circ Res.

[114] Helbling PM, Piñeiro-Yáñez E, Gerosa R, Boettcher S, Al-Shahrour F, Manz MG (2019). Global Transcriptomic Profiling of the Bone Marrow Stromal Microenvironment during Postnatal Development, Aging, and Inflammation. Cell Rep.

[115] Abegunde SO, Buckstein R, Wells RA, Rauh MJ (2018). An inflammatory environment containing TNFα favors Tet2-mutant clonal hematopoiesis. Exp Hematol.

[116] Liao M, Chen R, Yang Y, He H, Xu L, Jiang Y (2022). Aging-elevated inflammation promotes DNMT3A R878H-driven clonal hematopoiesis. Acta Pharm Sin B.

[117] Sanmiguel JM, Eudy E, Loberg MA, Young KA, Mistry JJ, Mujica KD (2022). Distinct Tumor Necrosis Factor Alpha Receptors Dictate Stem Cell Fitness versus Lineage Output in Dnmt3a-Mutant Clonal Hematopoiesis. Cancer Discov.

[118] Caiado F, Kovtonyuk LV, Gonullu NG, Fullin J, Boettcher S, Manz MG (2023). Aging drives Tet2+/− clonal hematopoiesis via IL-1 signaling. Blood.

[119] Boy M, Bisio V, Zhao LP, Guidez F, Schell B, Lereclus E (2023). Myelodysplastic Syndrome associated TET2 mutations affect NK cell function and genome methylation. Nat Commun.

[120] Campillo-Marcos I, Alvarez-Errico D, Alandes RA, Mereu E, Esteller M (2021). Single-cell technologies and analyses in hematopoiesis and hematological malignancies. Exp Hematol.

[121] Triana S, Vonficht D, Jopp-Saile L, Raffel S, Lutz R, Leonce D (2021). Single-cell proteo-genomic reference maps of the hematopoietic system enable the purification and massive profiling of precisely defined cell states. Nat Immunol.

[122] Izzo F, Lee SC, Poran A, Chaligne R, Gaiti F, Gross B (2020). DNA methylation disruption reshapes the hematopoietic differentiation landscape. Nat Genet.

[123] Heimlich JB, Bhat P, Parker AC, Jenkins MT, Vlasschaert C, Ulloa J et al. Mutated cells mediate distinct inflammatory responses in clonal hematopoiesis. bioRxiv 2022. https://www.biorxiv.org/content/10.1101/2022.12.01.518580v2.

[124] Wolock SL, Krishnan I, Tenen DE, Matkins V, Camacho V, Patel S (2019). Mapping Distinct Bone Marrow Niche Populations and Their Differentiation Paths. Cell Rep.

[125] Li H, Bräunig S, Dhapolar P, Karlsson G, Lang S, Scheding S (2023). Identification of phenotypically, functionally, and anatomically distinct stromal niche populations in human bone marrow based on single-cell RNA sequencing. Elife.

[126] Zhang J, Wu Q, Johnson CB, Pham G, Kinder JM, Olsson A (2021). In situ mapping identifies distinct vascular niches for myelopoiesis. Nature.

[127] Crosse EI, Gordon-Keylock S, Rybtsov S, Binagui-Casas A, Felchle H, Nnadi NC (2020). Multi-layered Spatial Transcriptomics Identify Secretory Factors Promoting Human Hematopoietic Stem Cell Development. Cell Stem Cell.

[128] Calvanese V, Capellera-Garcia S, Ma F, Fares I, Liebscher S, Ng ES (2022). Mapping human haematopoietic stem cells from haemogenic endothelium to birth. Nature.

[129] Oetjen KA, Lindblad KE, Goswami M, Gui G, Dagur PK, Lai C (2018). Human bone marrow assessment by single-cell RNA sequencing, mass cytometry, and flow cytometry. JCI insight.

[130] Lee NYS, Li M, Ang KS, Chen J (2023). Establishing a human bone marrow single cell reference atlas to study ageing and diseases. Front Immunol.

[131] Torlakovic EE, Brynes RK, Hyjek E, Lee SH, Kreipe H, Kremer M (2015). ICSH guidelines for the standardization of bone marrow immunohistochemistry. Int J Lab Hematol.

[132] Hernandez S, Lazcano R, Serrano A, Powell S, Kostousov L, Mehta J (2022). Challenges and Opportunities for Immunoprofiling Using a Spatial High-Plex Technology: The NanoString GeoMx® Digital Spatial Profiler. Front Oncol.

[133] Angelo M, Bendall SC, Finck R, Hale MB, Hitzman C, Borowsky AD (2014). Multiplexed ion beam imaging of human breast tumors. Nat Med.

[134] Stack EC, Wang C, Roman KA, Hoyt CC (2014). Multiplexed immunohistochemistry, imaging, and quantitation: A review, with an assessment of Tyramide signal amplification, multispectral imaging and multiplex analysis. Methods.

[135] Kennedy-Darling J, Bhate SS, Hickey JW, Black S, Barlow GL, Vazquez G (2021). Highly multiplexed tissue imaging using repeated oligonucleotide exchange reaction. Eur J Immunol.

[136] Kinkhabwala A, Herbel C, Pankratz J, Yushchenko DA, Rüberg S, Praveen P (2022). MACSima imaging cyclic staining (MICS) technology reveals combinatorial target pairs for CAR T cell treatment of solid tumors. Sci Rep.

[137] Weeks LD, Niroula A, Neuberg D, Wong W, Lindsley RC, Luskin MR et al. Prediction of Risk for Myeloid Malignancy in Clonal Hematopoiesis. NEJM Evid. 2023;2. 10.1056/evidoa2200310. 10.1056/evidoa2200310 PMC10361696 37483562

[138] Gu M, Kovilakam SC, Dunn WG, Marando L, Barcena C, Mohorianu I (2023). Multiparameter prediction of myeloid neoplasia risk. Nat Genet.

[139] Köhnke T, Majeti R (2021). Clonal Hematopoiesis: From Mechanisms to Clinical Intervention. Cancer Discov.

